# Power considerations for the application of detrended fluctuation analysis in gait variability studies

**DOI:** 10.1371/journal.pone.0174144

**Published:** 2017-03-21

**Authors:** Nikita A. Kuznetsov, Christopher K. Rhea

**Affiliations:** Department of Kinesiology, University of North Carolina at Greensboro, Greensboro, North Carolina, United States of America; University of Rijeka, CROATIA

## Abstract

The assessment of gait variability using stochastic signal processing techniques such as detrended fluctuation analysis (DFA) has been shown to be a sensitive tool for evaluation of gait alterations due to aging and neuromuscular disease. However, previous studies have suggested that the application of DFA requires relatively long recordings (600 strides), which is difficult when working with clinical populations or older adults. In this paper we propose a model for predicting DFA variance in experimental data and conduct a Monte Carlo simulation to estimate the sample size and number of trials required to detect a change in DFA scaling exponent. We illustrate the model in a simulation to detect a difference of 0.1 (medium effect) between two groups of subjects when using short gait time series (100 to 200 strides) in the context of between- and within-subject designs. We assumed that the variance of DFA scaling exponent arises due to individual differences, time series length, and experimental error. Results showed that sample sizes required to achieve acceptable power of 80% are practically feasible, especially when using within-subject designs. For example, to detect a group difference in the DFA scaling exponent of 0.1, it would require either 25 subjects and 2 trials per subject or 12 subjects and 4 trials per subject using a within-subject design. We then compared plausibility of such power predictions to the empirically observed power from a study that required subjects to synchronize with a persistent fractal metronome. The results showed that the model adequately predicted the empirical pattern of results. Our power simulations could be used in conjunction with previous design guidelines in the literature when planning new gait variability experiments.

## Introduction

Even seemingly perfectly periodic movements such as foot falls during human locomotion vary from one cycle to the next. In addition to characterizing the magnitude of this variability, identifying its temporal structure has become of interest in gait studies, as changes in variability structure may be an indicator of the adaptive capacity of the locomotor system [1, 2] and may help to reveal neural control strategies [3]. Detrended fluctuation analysis (DFA) [4] is a frequently used metric to characterize temporal structure in gait parameters, such as stride time. DFA has relatively low bias and variance compared to other fractal data analysis methods [5, 6], and is computationally straightforward. The outcome metric of the analysis is the scaling exponent—DFA α—which indexes the type of serial correlation present in the signal such that DFA α is 0.5 for uncorrelated series, between 0.5 and 1 for statistically persistent series, and between 0 and 0.5 for anti-persistent series [4, 7]. Previous experimental work has shown that young, healthy adults exhibit persistence in their stride time fluctuations, but aging or movement system pathology such Parkinson’s disease is associated with a shift toward uncorrelated behavior, potentially reflecting increased fall-risk [2].

The performance of DFA as a method to detect long-rage correlations in physiological data has been previously examined under the conditions of different trends in the data [8–10], noise and artifacts [5, 10], different data pre-processing filters [9], missing data [10, 11] and signal coarse-graining [12]. One practical limitation of DFA when applying it in the context of human movement studies is the amount of data required to accurately characterize the temporal structure of a time series. Previous studies focusing on variability of stride time in human locomotion suggested that continuous walking for approximately 12 min (600 strides at 1.2 s per stride) [13] or an average of three 6 min trials (300 strides) should be used for DFA analysis [14]. These studies were primarily focused on accurate identification of long-range correlations—a form of statistical dependence where the value of autocorrelation decays as a power law [7], with temporal correlations present over many hundreds of strides. However, numerical simulations also show that DFA can be successfully applied to correlated time series as short as 64 observations with the reservation the that the DFA α estimates become more variable, especially for persistent Gaussian noises [5], and are restricted to detecting short-range correlations. Short-range correlations are characterized by an exponentially decreasing autocorrelation, with temporal correlations present only over a small window of observations [5, 15]. Given that some clinical populations may not be able to walk for long periods of time at once, there is a need to develop guidelines on how to use DFA in experiments collecting short time series of gait variables such as stride time and length. Once these guidelines are developed, the value of examining short-range correlations in clinical or older populations can be studied to determine if similar or new information about neural control strategies can be discerned that were previously restricted to long-range correlation analysis.

Kirchner et al. [16] suggested that one solution to the short gait time series problem is to perform DFA on concatenated recordings. These authors ‘stitched’ together several very short bouts of overground walking consisting of 25 strides into one long time series and found that the DFA α value associated with the stitched time series still reliably distinguished inter-stride timing variability between healthy control subjects and individuals with Parkinson’s disease. These results are encouraging because they suggest that the detection of change in short-range statistical correlation may be sufficient to characterize differences in gait dynamics between two groups without having to document long-range correlations. However, this simple concatenation strategy may be problematic because it may introduce spurious correlation structure due to the edge effects at concatenation points, especially when concatenating very short time series with strong persistent correlations. The procedure also forces to examine the linear scaling in the DFA fluctuation function across scales that were not actually recorded experimentally (i.e., those longer than the number of strides within a trial). While a similar concatenation procedure has been applied to simulated signals without any apparent effect on DFA α estimates in recordings with persistent dynamics [10], one clear difference from Kirchner et al. [16] is that the simulated time series were very long (10^20^) and came from a single instance of a stochastic process that was cut into segments to be concatenated, a procedure different from generating multiple independent stochastic signals and stitching them together. This latter case is more similar to what happens when a subject repeats multiple separate bouts of walking, especially for overground locomotion because each trial has slightly different initial conditions and may led to different walking speed, stride length, and cadence.

An alternative strategy to avoid these issues with concatenation would be to record a greater number of trials of short duration, characterize DFA scaling in each trial, and then average across trial repetitions to increase the reliability of the DFA α estimate. To apply DFA successfully in this case, one would need to consider the statistical power of the design and the type of statistical tests used to compare the experimental conditions in the face of uncertainty of DFA α estimates. This uncertainty depends on the length of the time series, number of trials recorded, and number of subjects. Consideration of statistical power prior to conducting an experiment is important because underpowered studies overestimate the true effect size and are less likely to be reproducible [17].

The purpose of this paper is to provide a procedure for *a priori* power estimation when applying DFA to analyze relatively short time series of gait stride time (specifically, 100, 150, and 200 strides) when using two-group between- and within-subject designs. Differences in the short-range statistical correlations detected in these relatively short recordings may be sufficient to characterize differences in gait dynamics between two groups or experimental conditions as shown by Kirchner et al. [16]. The question we sought to answer was how many subjects and trials are required when researchers are limited to collecting only 100–200 strides per trial (this corresponds to 2 to 4 minutes of walking assuming stride time of 1.2 s). We approached this question by using Monte Carlo simulations to estimate power based on the expected sources of uncertainty in DFA α estimates. We then compared the simulation results with the observed power in a gait experiment that used metronome pacing to alter stride time variability structure. We hypothesized that simulation predictions would correspond to the observed power in the experiment and our results showed support for this hypothesis.

## Methods

### DFA analysis

The first step of the analysis is to integrate the measured time series, *x*, to obtain cumulatively summed signal $y_{N}=\sum_{i=1}^{N}\left[x_{i}-\hat{x}\right]$, where *N* is the length of the series and $\hat{x}$ is average of *x*. This step ensures that the original time series is converted into a random walk-like process. Importantly, cumulative summation is only required if the time series *x* is an instance of a random Gaussian process [18], which is the case for stride time.

In the second step, the signal is divided into *K* non-overlapping windows of length *w* and a linear least squares fit is applied within each window to obtain the local trend. Local variance around the trend is calculated as $y_{l}^{2}=\frac{1}{w}\sum_{i=1}^{w}{\left(y_{i}-\left(a*y_{i}+b\right)\right)}^{2}$, where *a* and *b* are the slope and the intercept of the local trend, *l* is the window number, and *i* is the particular sample measurement within the window and is reset to 1 at the beginning of each window. There are also several modifications of this step such as using overlapping windows or different polynomial orders for local detrending [19]. The detrended fluctuation function, *F*_*d*_*(w)*, is then calculated based on the average of the local variances across all *K* windows:

$$F_{d}\left(w\right)=\;\sqrt{\frac{1}{K}\sum_{l=1}^{K}y_{l}^{2}}$$ (1)

The last step is to characterize the scaling relation between the magnitude of detrended fluctuation on the window length. If there are no correlations in the original time series x, the relation between *F*_*d*_*(w)* and *w* is the same as for the standard random walk: *F*(*w*)~*w*^0.5^, indicating that the time series x is random. If α > 0.5, then there is persistence in the original time series such that distant observations have non-zero positive autocorrelation. If α < 0.5, then there is anti-persistence, such that there is alternation of larger- and smaller-than-average values in the original time series. There is a relation between the *F*_*d*_*(w)* and the autocorrelation function as described in Peng et al. [20] and more recently in Höll and Kantz [21].

To estimate the scaling exponent DFA α, the slope of the linear fit of log(*w*) vs. log(*F(w)*) is calculated. In a truly long-range correlated signal there is no characteristic correlation scale and linear scaling persists for at least 3 orders of magnitude in the DFA plots (10^1^, 10^2^, 10^3^) [20]. Long-range correlations are impossible to establish from concatenated sequences of short time series because the maximally resolvable correlation would be equal to the length of the concatenated time series.

Within the gait variability literature, a range of window sizes ranging from 16 to *N*/9 has been suggested by Damouras et al. [13] to estimate scaling properties when working with longer stride time recordings to increase between-subject consistency of DFA α estimates. However, the range of available window sizes is severely limited when using short time series. Therefore, we assumed a wider range of scales for DFA scaling: between 4 and *N*/4 observations. The minimum window length to estimate scaling should be 4 observations to ensure a robust fit of the local trend. The maximum length is typically set to be as least to *N*/4 because the estimate of *F*_*d*_*(w)* is based on too few observations if the window size is too large.

### Simulations for power analysis

Variability of DFA α within a single group of subjects (σ_overall_) was assumed to stem from three sources: σ_Overall_ = σ_Subject_ + σ_DFA_ + σ_Error_, where σ_Subject_ is variability due to inter-subject individual differences, σ_DFA_ is due to uncertainty of DFA α estimation for each subject (it depends on time series length and the number of trials used per subject), and σ_error_ is the uncontrolled experimental error per measurement.

**Fig 1:**
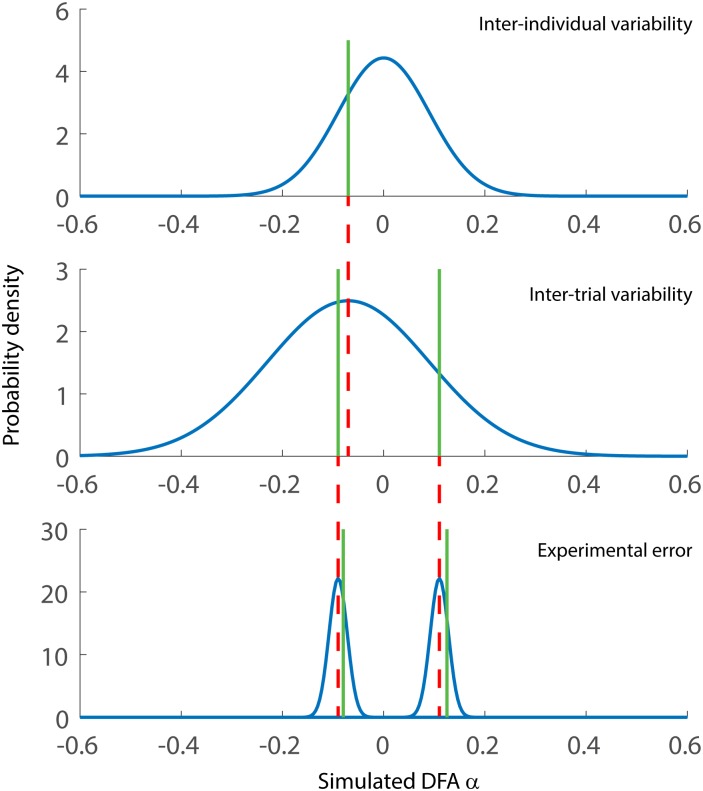
Illustration of the process for generating a simulated DFA α value for a single subject. A random number (green line) is drawn from a normal distribution with mean 0 and SD = 0.09 which represents expected inter-individual variability. Then two random numbers are drawn from the distribution centered on the individual deviation value (red dotted line) and SD = 0.16. This distribution simulates the expected variability of DFA α from trial to trial when using time series consisting of 100 data points (the SD of this distribution is smaller for longer time series). In this case, two trials are illustrated but we used 1, 2, 4, 6, and 8 trials in the full simulation. The experimental error is added to the trial values. in the final step of the simulation. The overall group value, *μ*, is then added to the average of the two resulting values to obtain the simulated DFA α value for a single individual. In the between-subjects version of the Monte Carlo simulations, two groups are simulated by drawing randomly from two independent normal distributions. In the within-subjects version, random numbers were drawn from a bivariate normal distribution ensuring correlation of 0.89 (see Method section).

Given these sources of variability, we simulated DFA α values that are likely to arise in gait variability experiments with the following design: Treatment (a = 2 levels, *μ*_*1*_ = 0.8 and *μ*_*2*_ = 0.70), Subjects (*N*, ranging from 3 to 50), and Trials per subject (*T*, ranging from 1 to 8). Magnitude of the treatment difference, *μ*_*1*_–*μ*_*2*_, was a fixed factor set at 0.10 (medium effect). Fig 1 presents the schematic of the procedure for the simulation.

Subject was a random factor assumed to be normally distributed with mean 0 and SD = 0.09 based on previous reports of individual differences in DFA α of stride time in healthy young adults during treadmill locomotion [22]. In the between-subjects version of the design, subject values in each treatment group were sampled from two independent normal distributions. In the within-subjects version, subject values in two groups were correlated by sampling from a multivariate normal distribution with covariance 0.0072 and variance 0.0081 (this variance matched the SD of the normal distributions in the between-subjects version), ensuring a correlation coefficient of 0.89.

The Trial factor was nested within each subject and was considered random under the assumption of that there is no practice effect. The estimated SD for this factor was 0.16, 0.12, and 0.10 to simulate DFA α variability that is expected when using trial lengths of 100, 150, and 200 strides, respectively. To estimate these magnitudes, we simulated 1000 fractional Gaussian noise (fGn) [15] time series with α = 0.9 per each trial length and calculated the SD of DFA α across the simulations. As shown in previous simulations [5] and in our own experience, the SD of DFA α increases for more persistent fGN signals. Hence, α = 0.9 was chosen because it represents the largest (and most conservative) estimate of variability compared to other α values. The fGn time series were simulated using the Davies and Harte algorithm [23]. The script for the simulation and the DFA α SD for a range of trial length is provided in S1 File.

Lastly, we assumed that each trial measurement is affected by an uncontrolled experimental error which was taken to be normally distributed with mean 0 and SD = 0.18 (equal to 20% of the between-subject variability).

To estimate statistical power to detect a given fixed effect, *μ*_*1*_–*μ*_*2*_, we simulated this design 5000 times for each combination of number of subjects and trials per subject. In order to simulate the effects of using different time series lengths, we used estimated SD of DFA α from the fGn simulations as the SD of the Trial factor (e.g. Trial SD = 0.16 for time series length of 100 points). We then applied *t*-tests (between- or within-subjects) to the simulated datasets and used the proportion of rejected null hypotheses (out of 5000) as an estimate of the predicted power. When there was more than one trial per subject, simulated DFA α values were averaged across trials for each subject prior to applying the *t*-tests. All computations were performed in Matlab 2015b (Mathworks, Natick, MA). The simulation script is provided in the supplement.

### Experimental data

We also re-analyzed experimental data from Rhea et al. [24] to evaluate the plausibility of the power predictions provided by the simulations. In that experiment healthy young adults (8 males and 7 females; age: 27.8 ± 4.4 yrs) continuously walked on a fixed-speed treadmill at a self-selected pace (1.3 m/s) for 15 minutes in one condition and in a separate condition subjects synchronized their strides with a persistent visual metronome (α = 0.98; CV = 6.15%). The visual metronome presented images of the left and right feet that appeared with a period prescribed by the persistent metronome. Subjects made approximately 800 strides in the self-paced condition and 750 strides in the synchronization condition. All procedures were approved by the Institutional Review Board at the Providence VA Medical Center and conformed to the principles expressed in the Declaration of Helsinki. All participants signed a written informed consent form. For a complete description of the experimental setup see Rhea et al. [24].

The original analysis of these data reported that DFA α in the self-paced condition was 0.77 ± 0.09 and 0.87 ± 0.06 in the metronome condition. To estimate the empirically obtained power for detecting this difference between the conditions when using different time series lengths, we re-analyzed these data using non-overlapping windows of 100, 150, and 200 strides from each subject’s complete stride time series (1, 2, 4, or 6 windows were used). DFA α values were calculated for each window and averaged together when more than one window was used. We then estimated how the observed power changed as a function of number of subjects by statistically comparing the two groups (self-paced vs. persistent metronome) using DFA α values of randomly selected subsets from the full dataset. These subsets consisted of sample sizes ranging from 3 to 13 subjects. We opted for resampling because there are many possible ways to select 3 subjects out of 15. For a given sample size, we resampled a random subset of subjects without replacement 1000 times and calculated the proportion of the subsamples for which we were able to reject the null hypothesis using a repeated-measures *t*-test. This proportion served as an estimate of the obtained power to detect the difference between the self-paced and metronome paced groups using that sample size. The procedure was similar to the Monte Carlo procedure we used in the power simulation such that it allowed us to generate an empirical power curve as a function of number of subjects.

## Results

**Fig 2:**
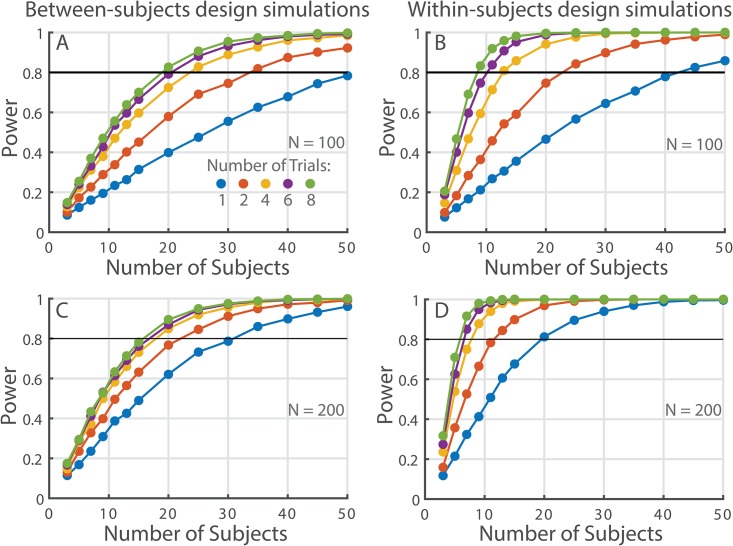
Simulation results. (A-B) These panels depict the expected power for between- and within-subject designs using 100 strides and an expected group difference of 0.1, corresponding to a medium effect size. (C-D) depict the same information for 200 strides per trial.

Simulation results allowed to visualize combinations of the number of subjects and number of trials sufficient to achieve adequate power to detect a medium effect size difference (*μ*_*1*_–*μ*_*2*_ = 0.10) when using between- and within-subject designs as illustrated in Fig 2. For example, simulation results showed that detecting a DFA α difference of 0.1 using a between-subjects design and 100 strides per trial would require at least 4 trials per subject and about 25 subjects per group to achieve 80% power and still be practically feasible in terms of data collection cost (yellow power curve in Fig 2A). When using a within-subjects design, the same effect could be detected when using either 25 subjects and 2 trials per subject or 12 subjects and 4 trials per subject (orange and yellow cures in Fig 2B, respectively). We also repeated the simulations for time series length of 200 samples. As expected, this simulation showed that the required number of subjects and trials to detect DFA α difference of 0.1 became smaller in both designs (Fig 2C and 2D). Interestingly, all of the simulations predicted a diminishing return on the power gained when adding more trials per subject. For example, there was a relatively large gain in power when using 4 trials per subject as compared to 2 in all simulation scenarios presented in Fig 2, but the gain in power was much lower when using 8 trials per subject as compared to 6.

**Fig 3:**
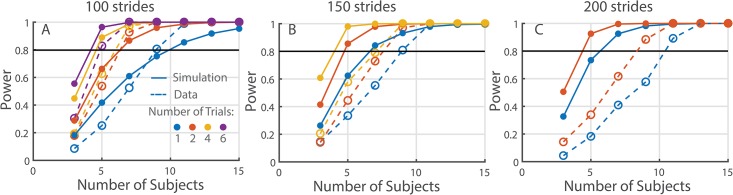
Comparison of power predictions (solid lines) with the experimentally observed power (dotted lines) for different trial lengths. (A) 100, (B) 150, and (C) 200 strides. Colored lines indicate the number of trials used per subject: 1 (blue), 2 (orange), 4 (yellow), and 6 trials (purple).

We then compared these power predictions to experimentally-observed power estimated from the fractal metronome study. We made power predictions for *μ*_*1*_ = 0.75 vs *μ*_*2*_ = 0.98. From our experience, typical DFA α values in the self-paced condition are around 0.75 on average, while the other prediction was made based on *a priori* consideration that subjects would produce the same structure in their stride time fluctuations as the structure prescribed by the persistent metronome (α = 0.98). We found that the simulated power was similar to the observed power when 100 strides were used for analysis (Fig 3A). However, as the number of strides increased from 150 to 200, simulation predictions became more liberal compared to the observed power results. For example, for 200 stride length simulation predicted that only about 6 subjects are needed to achieve 80% power when using one trial in a repeated-measures design, but the empirical result suggested that about 11 were really needed to detect the effect (Fig 3C).

**Fig 4:**
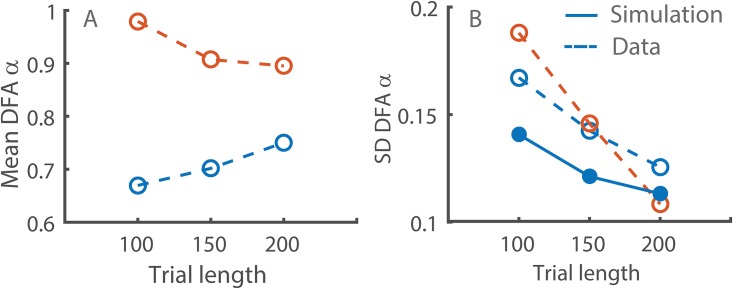
DFA α differences between the synchronization conditions as a function of trial length in experimental data. (A) Between-subject mean DFA α in the self-paced (blue lines) and persistent metronome (orange lines) conditions. (B) Standard deviation of DFA α in the same conditions. The solid blue line is the predicted between-subject variability from the simulation.

On further examination, the decreased match between the expected and obtained power was likely due to the discrepancy between the predicted and obtained sample group means. The mean DFA α values of the self-paced and fractal metronome conditions in the experiment were closest to the expected ones (0.75 vs. 0.98) when using trials of 100 strides, but the observed difference between the conditions became smaller when using longer trial lengths (see Fig 4A). As a result, the predicted and obtained DFA α values diverged, leading to a discrepancy in the expected and obtained power for longer trial lengths (150 and 200) presented in Fig 3B and 3C. At the same time, the power simulation captured the magnitude of inter-subject variability well, but slightly underestimated it for shorter time series lengths (Fig 4B). The simulation also successfully captured the increase in power that occurred with the inclusion of additional trials because the simulated power curves changed similarly to the experimental power as more trials were used in the analysis.

## Discussion

Power calculations reported in this paper extend previous guidelines provided for DFA use in gait studies [13, 14] by providing a procedure to estimate experimental power when applying DFA to short time series (100–200 strides) in the context of a two-group between- and within-subject designs. The type of design used in a study clearly affects its power [25], but no previous reports have explicitly considered this factor when providing guidelines for the application of DFA in gait variability studies. The results showed that our power simulation made reasonably accurate predictions for the pattern of empirically observed power and also adequately characterized between-subject DFA α variability level in the metronome synchronization experiment. The results showed that power predictions became more liberal compared to power obtained in the empirical data when using trials consisting of 150 and 200 strides, which was likely caused by the discrepancy between the predicted and empirical group means. The empirical group means were nearly identical to the predicted ones in the 100-stride trials, but became progressively more different from each other in the 150 and 200-stride trials. However, the between-subject variance was predicted well by the simulation, suggesting that it would be useful for study design planning.

Perrynowski et al. [14] suggested to use four 3-minute trials (150 strides if 1.2 sec per stride is assumed) in repeated-measures designs, but did not suggest any guidelines on the adequate number of subjects. Their recommendations were based on the criterion of reaching an intra-class correlation (ICC) of 0.8. These guidelines from Perrynowski et al. [14] could be used in conjunction with our power guidelines because the additional criterion of high ICC of the DFA estimates can help to select a particular combination of subject and trial numbers from the set of options presented by the simulation power curves. For example, our power simulations showed that to detect a difference of 0.1 and using 200 strides and four trials per subject would require about 8 subjects (Fig 2D; yellow line). Such design should produce adequate ICC values according to Perrynowski et al.’s suggestions. However, the design with only a single trial for each of the 20 subjects (Fig 2D; blue line) may not produce adequate ICC, and would be less adequate than the designs with more trial repetitions per subject.

It is important to note that power calculations are inherently predictive, so it may be difficult to test the accuracy of power predictions with respect to the limited set of empirical data unambiguously. Post-hoc power may not exactly match the prediction and has been criticized because it does not contribute new information to the completed results, but merely re-expresses the obtained *p*-values [26]. We attempted to get around this issue by considering the whole empirical power-curve for a variety of sample sizes. We did so by randomly resampling from the full dataset to provide an estimate for how empirical power changes as the number of subjects is decreased and compared this empirical power curve to the simulated power curve. This approach does require that there is a statistically significant group difference in the full data set and characterizes the deterioration of the probability to detect that difference as smaller size subsamples are selected. Despite the difficulty of ensuring predictive accuracy of the power estimates, rational *a priori* power analysis provides an initial guideline on the study’s likelihood to detect a given effect.

Our power simulations were based on several assumptions about the sources of variance in DFA α estimates. These assumptions should be met in a pilot study to maximize the predictive utility of the power simulations. First, we assumed that there are individual differences between subjects in terms of their stride time dynamics. This assumption is plausible given that such individual differences have been reported previously for finger tapping variability studies [27]—another seemingly periodic, yet variable behavior similar to gait because it similarly requires precise movement timing. Individual differences are also common in other types of motor behavior research [28]. A more challenging question is how to estimate the actual magnitude of the individual difference to ensure accurate power estimation. We used the value from a sample of healthy young adults reported by Choi et al. [22] who were walking on a speed-referenced treadmill at preferred pace. Under these conditions the standard deviation of DFA α between subjects was 0.09, but this value may be different when walking on a fixed-speed treadmill or in various clinical populations.

Second, we assumed that there were no practice effects for the repeated trials per subject such that DFA α variability from trial to trial was random. On a practical level, this assumption requires one to make sure that adequate practice is given prior to the experimental trials to minimize systematic changes in DFA α due to practice effects. Specifically, we assumed that the trial-by-trial DFA α fluctuations were characterized by the same normal distribution as that observed when simulating multiple instances of the fGn model for *H* = 0.9 (essentially equivalent to α = 0.9) based on the Davies and Harte algorithm [23]. A similar strategy was used by Delignieres et al. [5] when characterizing bias and variance of the DFA method as a function of time series length. If the fGn model is not a good fit for the stride interval series, then the estimates of the between-trial variance may be slightly inaccurate. However, the fGn model is always implicitly assumed when the DFA α is interpreted in terms of persistence, randomness, and persistence [29], so the wide use of this interpretation of the DFA results indicates that this assumption is widely accepted in the gait variability literature. At the same time, previous studies have shown that stride interval time series have multifractal features [30, 31], which may necessitate using simulation models capable of generating of multifractal time series when estimating the expected between-trial variability. But we believe that for the purposes of power estimation, the monofractal fGN model is a reasonable initial approximation. Especially given that multifractality is less likely to be apparent in very short time series. Instead of relying on the fGn model, we could have used empirical DFA α SD estimates provided by Damouras et al. [13]. These authors fit power law curves to the between-subject DFA α SD over the range of trial lengths from 256 to 900 strides using gait data from healthy young adults walking overground and on a treadmill. The values of expected DFA α variability obtained for overground and treadmill trials were slightly different and these differences could be incorporated to further adjust our power predictions. We opted for the simulated variance because we were interested in time series lengths shorter than 256 and also because empirically observed DFA α variability reflects not only trial-by-trial fluctuations, but also individual differences and experimental error. The shorter time series lengths we used are practical, if not required, in many clinical and aging populations.

Third, we also assumed that the DFA scaling can be adequately characterized by a single scaling exponent. Self-paced stride time dynamics are typically well-characterized using a single scaling region [1]. However, complex integrated physiological systems frequently show multiple scaling regions [32, 33]. Accordingly, it is important to examine raw DFA plots to ensure that scaling is present and that it is linear based on the pilot data. For example, when synchronizing to an external metronome, subjects tend to match the pacing of the persistent metronome better over long-term scales compared to short term-scales—a phenomenon known as complexity matching [34]. In principle, our power simulations can be easily adapted to focus on comparing the long-term scaling regions by decreasing the estimated DFA α variability related to trial-length (longer trials would have lower DFA variance). However, the performance of this prediction still needs to be evaluated.

Our paper does not deal with the extremely short time series consisting of fewer than 100 strides like the ones examined by Kirchner et al. [16] who used trials as short as 25 strides. Without concatenation DFA will not work well for such short time series because there is not enough data to calculate the scaling of variance, but there are some other methods that may apply to uninterrupted series as short as 50 strides such as the MLE estimate for the scaling exponent *H* [5], which is closely related to DFA α in terms of its interpretation. Another possibility could be to apply the magnitude and sign method proposed by Ashkenazy et al. [35]. This method has been suggested for the use on relatively “short” non-stationary signals [31].

We believe that simple concatenation of short trials is not appropriate because the physical interpretation of DFA α exponents using concatenated data is challenging. Concatenation increases the length of the time series and improves the reliability of linear regression estimate of the scaling exponent, but the correlation structure cannot be meaningfully interpreted beyond the scale associated with the window size used for concatenation [36]. For example, concatenating stride intervals in windows of 10 will allow to estimate correlations between stride 1 and 10, but correlation between stride 1 and 11, 12, 13 and so on is no longer resolvable. Accordingly, even if the time series is made artificially long, we cannot conclude anything about temporal correlations beyond the size of the window. Long-term recordings are required to unambiguously distinguish between exponential decay of the autocorrelation (indicative of short-range correlation) and power-law decay (indicative of long-range correlation) [7].

We illustrated our power estimation approach using a particular magnitude of the expected group DFA α difference, but the predictions can be easily simulated for other effect sizes using the Matlab functions accompanying the paper. The results presented in the manuscript are expected to be of relevance not only for gait dynamics but for locomotor control in general, where short records have to be carefully treated to extract physiologically meaningful results. For example, in recordings of wrist motion control based on actiwatch devices [37] or for quantifying change in long-range correlations and scaling of physical activity in sleep and circadian rhythm studies [38].

In conclusion, our simulations provided a reasonable estimate of obtained power in a behavioral gait experiment when using short time series of stride intervals. We suggest to use these predictions prior to conducing gait variability studies in conjunction with the guidelines of Perrynowski et al. [14]. A repeated cycle of prediction-observation using a variety of studies and manipulations will allow the scientific and clinical community to further determine the usefulness of our approach.

## Supporting information

S1 File Supplementary material. Supplementary material includes data and Matlab functions to reproduce all figures in the manuscript and stride time data from Rhea et al. (2014).
(ZIP)
