# Supplementary material for: Power considerations for the application of detrended fluctuation analysis in gait variability studies
Source: PLoS One. 2017 Mar 21;12(3):e0174144. doi: 10.1371/journal.pone.0174144 (PMC5360325; doi:10.1371/journal.pone.0174144)
Supplement: S1 File — Supplementary material includes data and Matlab functions to reproduce all figures in the manuscript and stride time data from Rhea et al. (2014). (ZIP) [file pone.0174144.s001.zip › Supplementary Material Description.docx]

**Supplementary Materials**

The supplementary materials contain the Matlab functions to create the data used for the figures and to reproduce the simulation results presented in the manuscript. If you have any questions, please contact Nikita Kuznetsov, Ph.D. at [nikita@uncg.edu](mailto:nikita@uncg.edu) or via ResearchGate profile: <https://www.researchgate.net/profile/Nikita_Kuznetsov2>.

**Main functions:**

1. **DFAPower_Between.m** – this Matlab script reproduces the Monte Carlo simulation results for the between-subjects design (Figures 2AC and 3). To reproduce Figure 2, please use the following parameters:

trials = [1,2:2:8];

subjectsN = [3:2:13 15:5:50];

Group1 = .8; % mean of group 1

Group2 = .7; % mean of group 2

var_subject = 0.09;

var_trial = 0.10;

var_error = var_subject*.2;

The raw data used to generate Figure 2 is also available in RawDataFigures.xls.

To reproduce Figure 3 simulation predictions:

trials = [1]; **- for single trial; [1,2] for two trials; [1,2,4] and [1,2,4,6] for 1-4 and 1-6 trials.**

subjectsN = [3:2:15];

Group1 = **.98;** % mean of group 1

Group2 = **.75;** % mean of group 2

var_subject = 0.09;

var_trial = **0.10**; **- edit to 0.16 for simulating results for trial length 100 points, 0.12 for 150 points, and .10 for 200 points.**

var_error = var_subject*.2;

The raw data used to generate Figure 3 is also available in RawDataFigures.xls.

1. **DFAPower_Within.m** – this Matlab script reproduces the Monte Carlo simulation for the within-subjects design (Figures 2BD and 3). To reproduce the results in these figures, use the same parameters as in the between-subjects simulation when running this script. The raw data used to generate Figures 2 and 3 is similarly available in RawDataFigures.xls.
2. **EmpiricalPower_Within.m** – this script reproduces the empirical power results presented in Figure 3 and 4. It uses the raw stride time data produced by subjects continuously walking on a fixed-speed treadmill at a self-selected pace (1.3 m/s) for 15 minutes in one condition and in a separate condition subjects synchronized their strides with a persistent visual metronome. To reproduce the empirical results presented in Figure 3, use the following inputs.

| Figure 3A | Figure 3B | Figure 3C |
| --- | --- | --- |
| WindowSize = 100;  WindowN = [1,2,4,6] | WindowSize = 150;  WindowN = [1,2,4] | WindowSize = 200;  WindowN = [1,2] |

The resulting empirical power values from this script are used to generate Figure 3 (using the values from the prw_curve variable in the function). The raw power curve data generated by the function are available in RawDataFigures.xls. This function also produces data to reproduce Figure 4 (stored in the resultOut variable). The data for Figure 4 is also available in RawDataFigures.xls.

1. **fGN_LengthSimulation.m** –script simulating the DFA α variance from trial to trial. BetweenTrialDFAVariance.xls – table reporting the standard deviation of DFA α estimates for simulated fGN time series of different lengths. These results are produced in the fGN_LengthSimulation script.

Figure 1 serves to illustrate our sampling procedure in the DFA variance model and does not contain any real.

**Supplementary functions:**

**fgnSim.m** – function to simulate fractional Gaussian noise.

**dfa.m** – function to perform the DFA analysis.

All scripts and functions were tested on Matab 2015b (MathWorks, Natick, MA).
